# Persistence, Seasonal Dynamics and Pathogenic Potential of *Vibrio* Communities from Pacific Oyster Hemolymph

**DOI:** 10.1371/journal.pone.0094256

**Published:** 2014-04-11

**Authors:** Carolin C. Wendling, Frederico M. Batista, K. Mathias Wegner

**Affiliations:** 1 Alfred Wegener Institute, Helmholtz Centre for Polar and Marine Research, Wadden Sea Station Sylt, Coastal Ecology, List, Schleswig-Holstein, Germany; 2 Instituto Português do Mar e da Atmosfera, Estação de Moluscicultura de Tavira, Olhão, Portugal; State Key Laboratory of Pathogen and Biosecurity, Beijing Institute of Microbiology and Epidemiology, China

## Abstract

Bacteria of the genus *Vibrio* occur at a continuum from free-living to symbiotic life forms, including opportunists and pathogens, that can contribute to severe diseases, for instance summer mortality events of Pacific oysters *Crassostrea gigas*. While most studies focused on *Vibrio* isolated from moribund oysters during mortality outbreaks, investigations of the *Vibrio* community in healthy oysters are rare. Therefore, we characterized the persistence, diversity, seasonal dynamics, and pathogenicity of the *Vibrio* community isolated from healthy Pacific oysters. In a reciprocal transplant experiment we repeatedly sampled hemolymph from adult Pacific oysters to differentiate population from site-specific effects during six months of *in situ* incubation in the field. We characterized virulence phenotypes and genomic diversity based on multilocus sequence typing in a total of 70 *Vibrio* strains. Based on controlled infection experiments we could show that strains with the ability to colonize healthy adult oysters can also have the potential to induce high mortality rates on larvae. Diversity and abundance of *Vibrio* varied significantly over time with highest values during and after spawning season. *Vibrio* communities from transplanted and stationary oysters converged over time, indicating that communities were not population specific, but rather assemble from the surrounding environment forming communities, some of which can persist over longer periods.

## Introduction

Bacteria of the genus *Vibrio* are highly abundant in the marine environment and include several pathogenic strains that can harm invertebrates and vertebrates, including humans [1]. In the aquaculture industry, mass mortalities resulting from *Vibrio* infections provoke severe production losses with great economic impacts in shrimp [2], [3], [4], [5], fish [6] and shellfish [7], [8], [9], [10] including the Pacific oyster *Crassostrea gigas* (Thunberg, 1793). The Pacific oyster is an important aquaculture species of great economic value with a production expanding more than six million tons per year [11]. Since the 1960's abnormal mass mortalities of the Pacific oyster, known as summer mortality syndrome (SMS), have been increasingly reported worldwide, including Japan [12], the United States [13], [14], France [15] and the Southern part of the North Sea [16]. Especially since 2008, reported mass mortalities of Pacific oyster spats increased dramatically in warmer seasons in some areas of France and New Zealand [17]. Multiple stressors such as elevated temperature, low dissolved oxygen, and limited energy resources after spawning were often associated with these reported mortality outbreaks [13], [18], [19], [20], [21], [22]. In combination with these factors bacteria of the genus *Vibrio* spp. have repeatedly been implicated as one causative agent [8], [9], [10], [13], [18], [19], [20], [21], [23], [24], [25], [26], [27] including recent adult mortalities in France associated with high abundance of *V. aestuarianus* [9], [26].

*Vibrio* spp. are ubiquitous in marine ecosystems and can utilize different lifestyles including free living, mutualistic, opportunistic or pathogenic forms [28], [29]. Pathogenicity of *Vibrio* is tightly linked to seawater temperature, which can on the one hand increase host susceptibility by weakening host's immune systems [30]. On the other hand high temperatures can result in a higher transmission and proliferation rate of *Vibrio* spp. or/and in an upregulation of several virulence factors involved in motility, host degradation, secretion, and antimicrobial resistance [31]. For instance, only at high temperatures, *Vibrio shiloi* can lyse the symbiotic zooxanthellae thereby causing severe coral bleaching events [32].

Bacteria of the genus *Vibrio* have been isolated and characterized from healthy and moribund *C. gigas* for more than 30 years [8], [9], [24], [27], [33], [34]. The predominant species associated with oyster mortality outbreaks were *V. splendidus*, *V. anguillarum*, *V. tubiashii* and *V. aestuarianus* [8], [9], [24], [27], [33], [34], [35]. The cultivable *Vibrio* flora harbored in healthy *C. gigas* is hypothesized to represent a bivalve specific community [36] with a high genetic diversity [37], [38], [39] ranging from 10^2^ to 10^4^ CFU/mg tissue with higher loads at higher temperatures [26].

Although, these findings have made great advances in characterizing pathogenic *Vibrio* spp. by isolating strains from moribund oysters, a comprehensive overview of the *Vibrio* community structure of healthy *C. gigas*, as well as an in-depth knowledge of its persistence over time is lacking.

Here, we describe the temporal dynamics of the *Vibrio* community structure in Pacific oysters stemming from two oyster beds that have been spared from mass mortalities thus far. Previous research showed that these oyster beds were genetically differentiated from each other and showed a bed specific response of the total bacterial community to disturbance [40]. Based on the genetic differentiation between beds we now wanted to disentangle the influences of host genetic bed affiliation and environmental site conditions on the *Vibrio* community by conducting a reciprocal transplant experiment. By means of repeated hemolymph sampling we were able to describe the persistence, dynamics and diversity of the *in situ Vibrio* community able to colonize *C. gigas* hemolymph during a six-month period. We further complement this data with an empirical assessment of the pathogenicity of local strains under elevated summer temperatures using controlled infection experiments on oyster larvae.

## Materials and Methods

Permission to collect oysters was given by the Nationalparkamt Schleswig-Holstein; http://www.nationalpark-wattenmeer.de/.

### Reciprocal transplant, hemolymph sampling, enumeration and isolation of *Vibrio spp.*

In May 2010 we collected 96 Pacific oysters *C. gigas* from two natural intertidal soft-bottom oyster reefs, i.e. Oddewatt (OW) (55°02′17″N, 08°26′32″E) and Diedrichsenbank (DB) (55°02′31″N, 08°26′53″E) from the Königshafen on the island of Sylt, North Sea, Germany. This area is characterized by a tidal regime (about 180 cm) and temperatures ranging from 0°C in winter to a maximum of 21.67°C in summer. We randomly collected healthy oysters with an average size of 7.41 cm±0.97 (mean ± SE). Oysters were brought to the lab and a hole was drilled with a small hand drill in the upper shell close to the adductor muscle through which a needle could be inserted to sample hemolymph from the adductor muscle. Afterwards oysters were individually labeled and deployed outside in the following manner: 48 oysters originating from each bed were equally deployed at OW and DB. This resulted in four different transplant groups, i.e. OW-OW, OW-DB, DB-OW, DB-DB (origin-site) containing 24 oysters each. Marked oysters were kept in plastic mesh bags (4 oysters per bag representing each transplant group) and anchored to sandy mud flats surrounded by the original oyster beds in the intertidal.

From May 2010 before deployment until October 2010 hemolymph was repeatedly sampled from each oyster once a month. At each time point we checked the oysters for mortality. Dead animals were denoted and removed from the batches. Hemolymph was withdrawn from each oyster with a 2-mL syringe attached to a needle of 23G×5/4″ that was inserted through the predrilled hole in the shell into the muscle tissue. During the sampling time in the field, withdrawn hemolymph was stored on ice. In the lab 4 µl of undiluted hemolymph was spread on *Vibrio* selective Thiosulfate Citrate Bile Sucrose Agar (TCBS) plates (Fluka Analytica, Sigma-Aldrich, Steinheim, Germany). Plates were incubated at 25°C for 24 hours. Afterwards colony forming units (CFU) were counted for each oyster and a random subset of 11–24 colonies (depending on growth of colonies) per month covering all transplant groups was taken for further analysis. Single colonies were randomly picked from the agar plates and resuspended in 3 mL Nutrient solution (5.0 g peptone, 3.0 g meat extract, 1.5 g NaCl in 1000 ml distilled water) and cultured at 25°C under constant shaking for 24 hours. We choose 25°C for isolation, because our main goal was to assess the pathogenicity of isolated strains at high summer temperatures and did not want to introduce bias by isolating strains at ambient temperatures observed in the field. This liquid bacteria culture was used for direct amplification of three different genes (16S rRNA, *PyrH* and *GyrB*) to determine the genetic affiliation (phylotype) based on multilocus sequence types (MLST) of the *Vibrio* cultures. The remaining cultures were cryopreserved at −80°C in 50% glycerol until further use.

### PCR and Gene sequencing

To increase the resolution of *Vibrio* isolate identification, we chose a MLST approach based on three different genes, i.e. *16S rRNA*, *GyrB* and *PyrH* (Primer details listed in supporting information: Table S1). Partial 16S rRNA gene of representative isolates was amplified directly from the liquid bacteria culture without prior extraction using universal primers 16S-27f and 16S-1392R. PCR mixtures were composed of 4 µL PCR buffer (Promega, Mannheim, Germany), 1.2 µL MgCl_2_(25 mM), 1.0 µL dNTPs (10 mM), 1.0 µL each forward and reverse primers (50 µM), 0.05 µL *Taq* polymerase (Promega, Mannheim, Germany) and 1 µL template DNA in a total volume of 20 µL. The amplification program used was one cycle at 95°C for 5 min, 30 cycles at 95°C for 30 s, 50°C for 30 s, 72°C for 90 s and one final cycle at 72°C for 10 min. Prior to amplification of the protein encoding genes (*PyrH and GyrB*), *Vibrio* cells were lysed for 3 min at 100°C. DNA amplification of *PyrH* and *GyrB* followed the protocol described by [41]. The PCR was conducted in a final reaction volume of 50 µL containing 5 µL Buffer (Peqlab, Erlangen, Germany), 1.0 µL dNTPs (10 mM), 1.0 µL each forward and reverse primers (50 µM), 0.5 µL *Taq* DNA polymerase (Peqlab, Erlangen, Germany), and 5 µL template DNA. The thermal program consisted of: (i) 5 min at 95°C; (ii) 3 cycles of 1 min at 95°C, 2 min 15 s at 55°C and 1 min 15 s at 72°C; (iii) 30 cycles of 35 s at 95°C, 1 min and 15 s at 55°C and 1 min and 15 s at 72°C; and (iv) a final extension for 10 min at 72°C. PCR purification and standard Sanger sequencing was performed by the Institute for Clinical Molecular Biology (IKMB), Christian-Albrechts-University Kiel.

### Controlled infection experiments

#### Larvae rearing

In order to estimate the pathogenic potential of isolated *Vibrio* strains we conducted controlled infection experiments on 11 day old larvae. Parental oysters were randomly collected from the Oddewatt (OW). Crosses were performed by strip-spawning 3 females and 3 males, resulting in three independent full-sib families. Gametes were stripped directly from the gonads and collected into 0.45 µm filtered, UV treated seawater. Fertilization was performed at a ratio of 200 spermatozoa per oocyte, with 4×10^5^ oocytes per family. After 20 min, spermatozoa were removed by collecting oocytes on a 20 µm mesh screen. One hour after fertilization embryos were transferred to the rearing tanks at a concentration of 5 embryos/ml. Larvae were kept at 21°C with salinity of 28 psu in 2L rearing tanks filled with 0.45 µm filtered, UV treated seawater and fed with *Isochrysis galbana* at a concentration ranging from 10 to150 cells/µL depending on larval age. Water was exchanged every second day. At day 10 after fertilization, families were pooled with equal contribution of each family.

#### Experimental infections

Out of the 93 sampled isolates, we were able to successfully sequence all genes needed for MLST genotyping in 74 isolates. Out of those we were able to grow 70 isolates under agitation at 25°C in Nutrient solution 1.5% NaCl for 20 h. We determined the bacterial concentration by optical density (OD) at 550 nm. We assessed the correlation between OD at 550 nm and colony forming units of 10 selected strains (details listed in supporting information: Figure S1) and observed that an OD of 1 at 550 nm corresponded to 4.63–5.24×10^8^ CFU/ml. Therefore, we used an approximation of an OD value of 1 to correspond to 5×10^8^ CFU/ml as has been previously done by [42]. Bacteria cells were centrifuged at 5500 rpm at 25°C for 5 min and resuspended in Nutrient agar at 1×10^9^ cells/mL. Controlled infection experiments on larvae were carried out using sterile 96-well culture plates, following a modification of the protocol described by [33]. Briefly, 10–15 larvae per family were placed in one micro-well containing 0.45 µm filtered and UV treated seawater. Larvae were challenged by bathing in a bacterial suspension containing 10^7^ cells/mL of each respective *Vibrio* isolate. We added PBS to 4 additional wells, serving as controls. Each experimental group was assayed in duplicates. Infection experiments were carried out in a constant climate chamber at 21°C. We considered 21°C as an optimal temperature to test *Vibrio* pathogenicity on oyster larvae for two reasons (1) *Vibrio* induced mass mortalities as well as oyster larvae only occur at warm summer temperatures (above 19°C), (2) 21°C is the maximum temperature found in the study area and projections of future sea surface temperatures do suggest that similar temperatures can be expected on average during summer months in the study area. Survival was observed at day 3 after inoculation using an inverted microscope by counting the amount of dead larvae, i.e. empty shells or closed larvae without velum movement and signs of necrosis.

### Data Analysis

#### Dynamics

There was no effect of oyster origin (factorial repeated measures ANOVA: χ^2^_(df = 6)_ = 0.43, p = 0.51), transplanted site (χ^2^_(df = 7)_ = 0.18, p = 0.67), as well as no significant interaction of origin and site (χ^2^_(df = 8)_ = 1.27, p = 0.26) on the amount of colony forming units (CFU). Therefore we pooled the data and conducted a multilevel linear model to assess differences in CFU between months, with CFU as the dependent variable and months as the independent variable. Significant effects were further analyzed with a Tukey HSD post-hoc test. Potential associations between CFU and temperature were analyzed with Pearson's correlation analysis.

#### Phylogenetic Analysis

Sequences were manually edited and automatically assembled using Sequencher™ 4.8 software. Edited gene sequences obtained from each single gene were compared against published sequences in NCBI GenBank using BLASTN algorithm with default settings based on 99% sequence identity to identify putatively close phylogenetic relatives of isolated *Vibrio*. Multiple alignment of concatenated sequences was created by MUSCLE, version 3.8.31 [43]. This included 2,560 positions after manual removal of ambiguous positions using JalView, version 2.8 [44]. We used jModelTest [45], [46] to select the best fitting substitution model of sequence evolution following recent recommendations [47]. Based on the concatenated sequences a phylogenetic tree was constructed using maximum-likelihood phylogenies PhyML v.3.0 [46], [48] by using the *General Time Reversible* model plus a discrete γ-distribution to account for rate heterogeneity among sites plus invariant sites (GTR+G+I) [49] as suggested by jModelTest. The following parameter settings were used for phylogenetic analysis: Transition/transversion ratio was estimated by PhyML; four gamma rate categories were used; a BIONJ tree was initially used; branch length and tree topology were optimized by PhyML using *Allovibrio fischerii* as an outgroup. Additionally we included 14 reference strains (Supporting information: Table S2) that were accessed from NCBI GenBank. Reliability of topology was assessed by bootstrapping with 500 replicates [50]. A radial cladogram was drawn using the Interactive Tree of Life web service at http://itol.embl.de/ [51], [52]. All sequences were deposited at GenBank with Accession Numbers KJ507408–KJ507477 (16 s), KJ507478–KJ507547 (GyrB), KJ507548–KJ507617 (PyrH).

#### Diversity analysis

To describe the diversity of the *Vibrio* community we first built a distance matrix based on the concatenated sequences using the DNADIST program within the PHYLIP software package based on the Kimura-2-parameter distance method with all other options set to default [53]. This distance matrix was imported into the MOTHUR software version 1.23.6 [54] to assign operational taxonomic units (OTUs) defined by 99% sequence similarity using the average neighbour algorithm. We calculated non-parametric OTU-based richness estimators (Chao1 and Shannon-Wiener Index) to estimate the diversity of the *Vibrio* community for the different sampling sites and sampling months. We further conducted a Pearson's correlation analysis to identify possible associations between diversity and temperature. Because *Vibrio* diversity correlated positively with mean temperature per sampling month we conducted a lag analysis to assess whether the *Vibrio* community composition reacts to quick temperature shifts or is stable over longer time periods. We sequentially extended the time window by 4 days in which temperature was measured starting from sampling date for each month, calculated the average temperature for each time window and correlated it with the respective diversity index. The highest correlation coefficient over all months can then give an idea whether community composition is integrated over longer time periods or reacts quickly to shifts in temperature.

#### Persistence

Temporal and spatial differences as well as the persistence of the *Vibrio* community structure between the different sampling months, as well as between transplanted and original oysters were analysed using UniFrac distances, a metric of the unique phylogenetic distance between communities, available as a web application at http://bmf.colorado.edu/unifrac [55], [56], [57]. UniFrac was applied to cluster the sequences from the different transplant groups by months as well as by site and origin and to test which months and transplant groups were significantly different from each other using UniFrac-test and Principal Coordinate Analysis (PCoA). P-values have been corrected for multiple comparisons using Bonferroni correction.

#### Pathogenicity

Virulence of a given strain was assessed as induced mortality at day 3 after inoculation. Virulence was not normally distributed as determined by the Shapiro-Wilk test. Differences in induced virulence between the different clades as well as differences in average virulence per month were analyzed with non-parametric Kruskal-Wallis rank sum test. Possible influences of temperature and diversity (Chao1 and Shannon-Wiener Index) on mean and maximum virulence were assessed with a Spearman rank correlation.

Maximum virulence per sampling month correlated positively with temperature (Spearman rank correlation: r = 0.77, p = 0.08). To predict the probability of encountering a highly virulent strain per month we used a logistic regression, predicting the occurrence of strains with higher than average virulence (outliers in Fig. 5) by mean monthly temperature.

We estimated the variability within the duplicates per strain by calculating the mean difference between all duplicates and the variance (mean = 4.61, variance = 29.38). In addition we estimated the mean difference of a total of 1000 permutations of randomly 70 chosen differences between the two datasets and calculated the average variance (mean = 16.81, variance = 40.13). With that we could show, that variability within the experimental treatment was low, and results are reliable.

All statistical analyses were performed in the R 2.15.2 statistical language (R Development Core Team, 2011).

## Results

### Abundance

**Figure 1:**
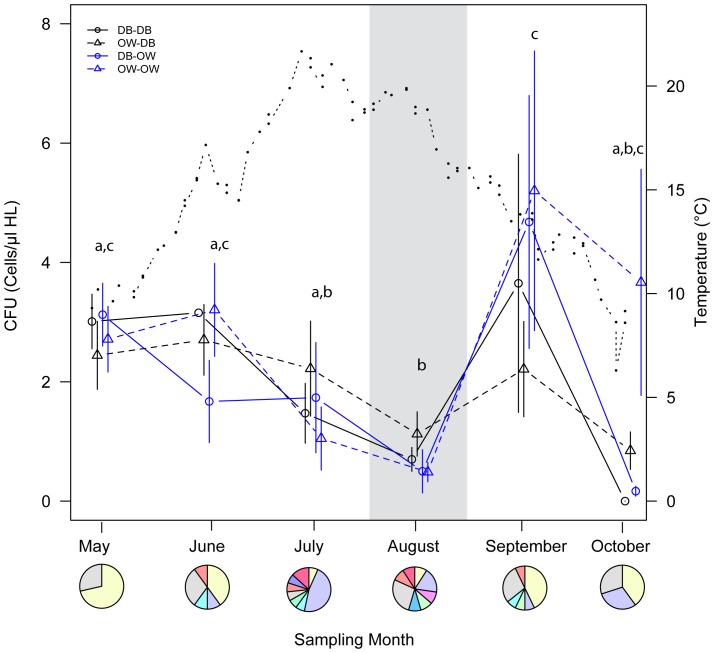
Temporal variation in the amount of *Vibrio* bacteria in oyster hemolymph (HL). Quantification of total *Vibrio* spp. (open circles and triangles) isolated from oyster hemolymph stemming from the four transplant groups (origin_site), i.e. DB_DB, DB_OW, OW_DB, OW_OW. Blue lines represent oysters assayed in site OW and black lines represent oysters assayed in site DB. Solid lines show oyster origin DB, while dashed lines show oyster origin OW. Water temperature is shown on the secondary y-axis (full circles, dotted line). The area shaded in grey marks the spawning period. Different letters indicate significant differences according to Tukey HSD post hoc test. Pi charts show relative proportion of *Vibrio* isolates by phylogenetic association based on 99% similarity by BLASTN analysis in each sampling month. Color codes correspond to those in Figure 2.

Temporal variation in the amount of *Vibrio* bacteria in oyster hemolymph was monitored from May to October 2010 by counting colony forming units (CFU) on a monthly basis. Cultivation of *Vibrio spp.* from oyster hemolymph revealed that *Vibrio* are consistently present as a member of the oyster hemolymph community. *Vibrio* abundances varied significantly over time (multilevel linear model: χ^2^_(df = 9)_ = 23.05, p<0.001) but showed similar patterns in all transplant groups. The number of cultivable *Vibrio* in the oyster hemolymph decreased from May to August, followed by a drastic increase after the spawning period (i.e. in September with a maximum value of 5.2 CFU µl^−1^) and a decrease towards autumn when temperature dropped down (Figure 1). Total bacteria load was not significantly correlated to temperature (Pearson's correlation: r = −0.19, t_(df = 4)_ = −0.39, p = 0.72) indicating that temperature was not the main driver for *Vibrio* load in oyster hemolymph.

### Diversity

**Figure 2:**
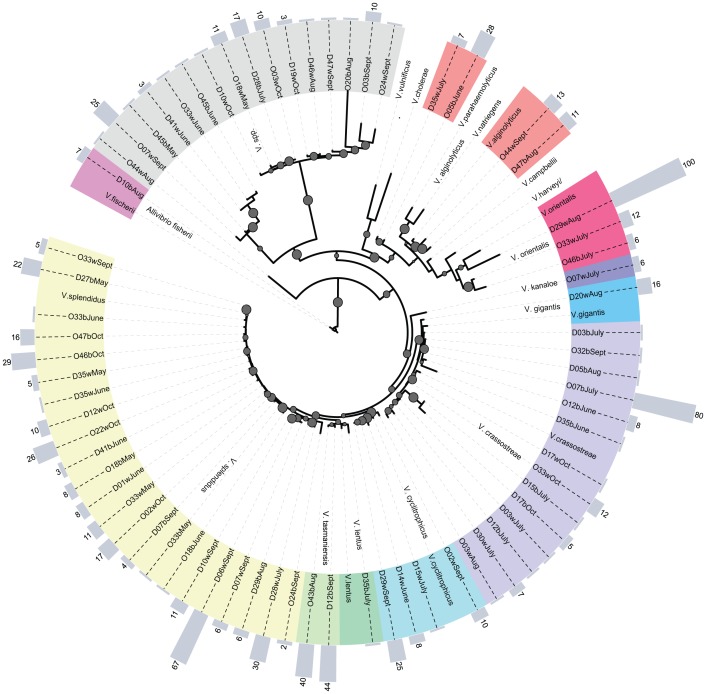
Phylogenetic relationships of *Vibrio* isolates sampled from oyster hemolymph. Maximum likelihood method (GTR+G+I substitution model) using concatenated sequences of three genes (16S rRNA, *GyrB*, *PyrH*) (2560 bp). Bootstrap percentages above 80% are represented by grey dots at the parent nodes. Virulence of each strain is depicted by the grey bars on the outer ring (a value of 100 corresponds to 100% mortality during inoculation experiments). Assignment to the different clades (see legend) based on 99% sequence similarity by BLASTN analysis is depicted in color on the inner ring.

From April to October 2010 we isolated a total of 93 strains from the hemolymph of *C. gigas*. Out of these we successfully determined the virulence and MLST genotype for 74 strains. Based on BLASTN comparison of each single gene the phylogenetic affiliation of 16 strains could not be assigned uniquely to one clade within the genus *Vibrio* and are therefore named as *Vibrio sp*. All other strains were assigned to 10 different *Vibrio* species that fell into four distinct clades as defined by [58], [59] (Figure 2). Strains of the *Splendidus* clade were the most frequently observed (64.3%) and were represented by seven different species: *V. splendidus*: 23 isolates, *V. crassostreae:* 14 isolates, *V. cyclitrophicus*: 3 isolates, *V. tasmaniensis:* 2 isolates, *V. gigantis*, *V. lentus* and *V. kanaloae*: 1 isolate each. The second most common clade was represented by members of the *Vibrio* core (*Harveyi* clade), with a total of 4 isolates (i.e. 5.7%), all represented by *V. alginolyticus*. We further assigned three isolates to the *Orientalis* clade (4.29%), and another one single isolate to the *Fischeri* clade, identified as *Allivibrio fischerii*. Of all isolated species, *V. splendidus* was the only one that was consistently present in oyster hemolymph in every month.

**Figure 3:**
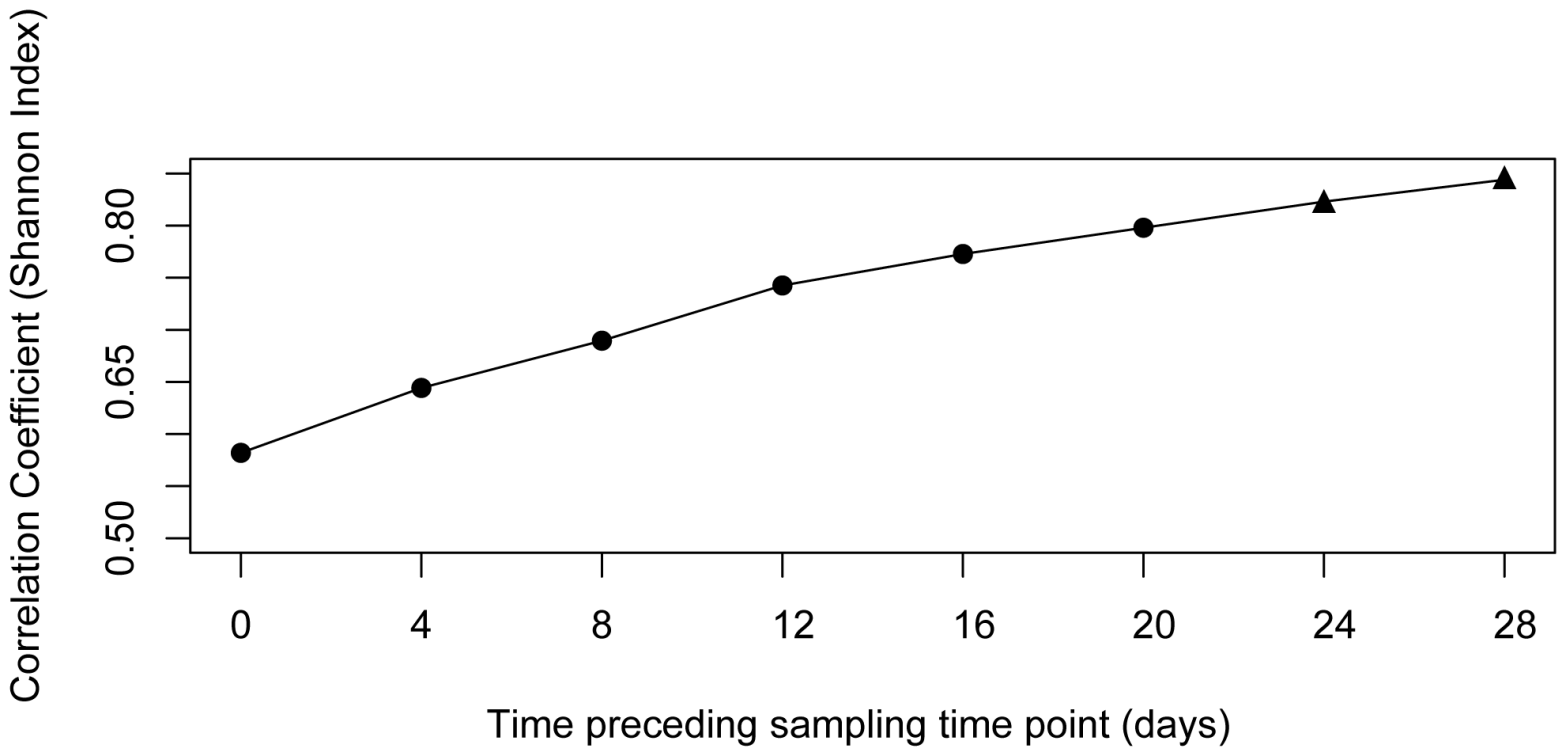
Lag analyses for the correlation between diversity (Shannon Index) and mean temperature integrated over 28 days prior to the sampling event. Day 0 corresponds to the temperature measured at the sampling day representing an immediate response, while day 28 uses the mean temperature of the previous month representing a slower response to temperature shifts. Significant correlations between diversity and temperature are depicted by black triangles.

Analysis of operational taxonomic units (OTUs) based on concatenated MLST sequences resulted in 41 OTUs on the basis of 99% sequence similarity (Table 1). The *Vibrio* community structure differed significantly between months (Figure 1, UniFrac significant test after Bonferroni correction: p<0.001). Total number and diversity was highest during summer (July–September) and lowest in May (Table 1). *Vibrio* communities showed a clear differentiation between spring/autumn (May, June/October) and the warm summer months (July, August, September, Figure 1). *Vibrio* diversity was positively correlated with mean temperature per sampling month (Chao1 index: r = 0.81, t_(df = 4)_ = 2.78 p = 0.05; Shannon-Wiener Index: r = 0.87, t_(df = 4)_ = 3.45 p = 0.03) and lag analysis revealed that this correlation was strongest when integrated over longer time periods as opposed to immediate responses to temperature shifts (Figure 3). Both sampling sites (DB and OW) had almost equal numbers of OTUs and shared nine OTUs. Richness estimators indicated that both sampling sites had a similar diversity (Table 1).

**Table 1:** Number of OTUs and richness estimators (Chao1 and Shannon-Wiener Index) for each sampling month (May–Oct) as well as at both sampling sites (Dietrichsenbank and Oddewatt) on the basis of 99% identity cut-off.

Group	No. of sampled colonies	No. of sequences	Richness estimators		
			No. of observed OTUs	Chao1	Shannon
DB	55	33	24	94	2.9
OW	58	37	26	73.5	3.1
May	11	7	4	7	1.15
June	16	12	9	19.5	2.09
July	24	14	12	27	2.44
Aug	24	11	11	66	2.39
Sept	23	15	12	24.5	2.39
Oct	15	11	7	12	1.77

### Persistence

**Figure 4:**
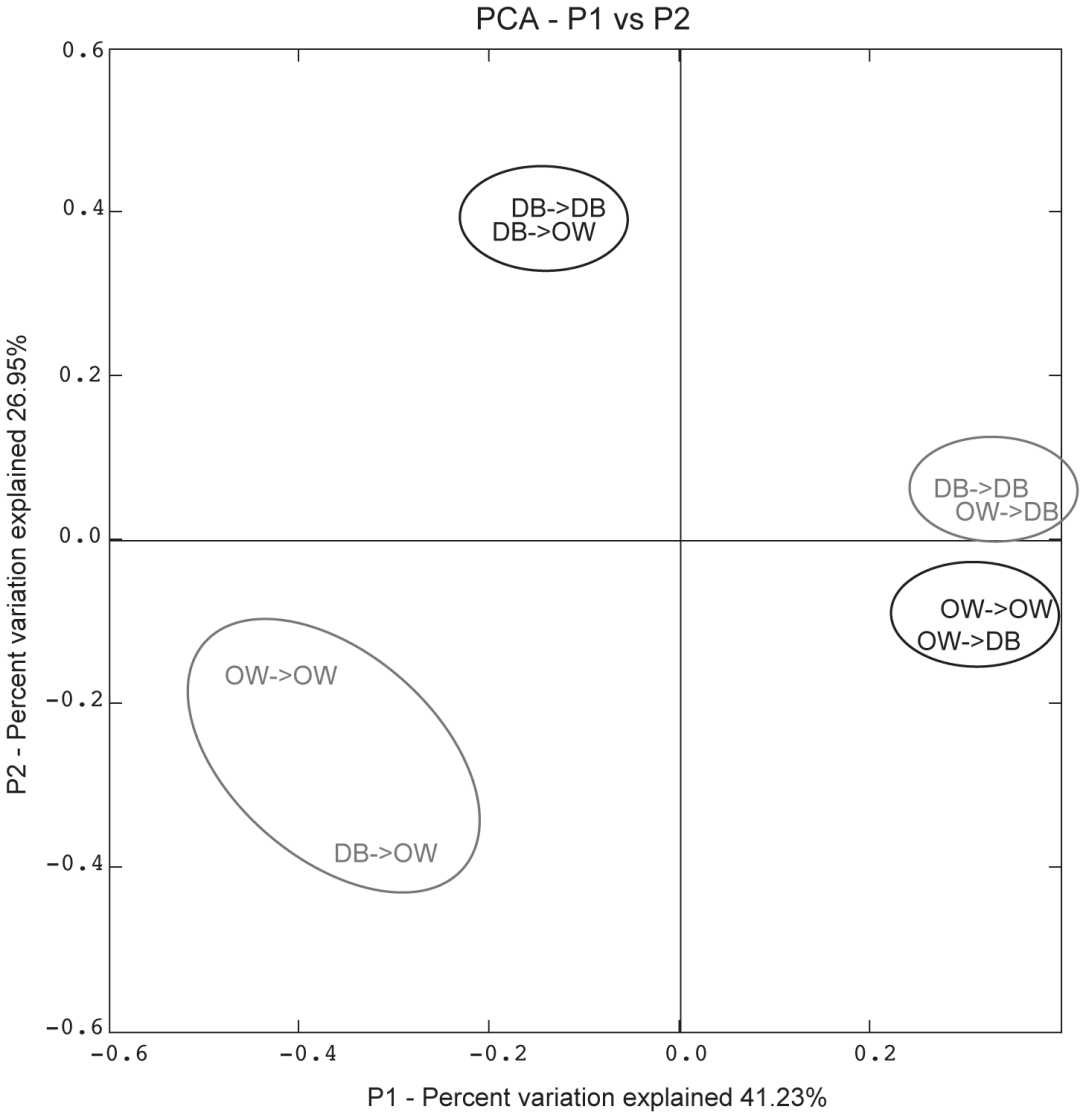
Ordination of *Vibrio* communities from all different transplants. Origin_Transplant site: DB->DB, DB->OW, OW->DB, OW->OW at the beginning (June in Black) and end (September in Grey) of the experiment. Shown are the first two principle coordinates from PCoA implemented in web-based UniFrac analysis.

To assess temporal and spatial variation in the *Vibrio* community structure between original and transplanted oyster samples we used June as the starting month to give the *Vibrio* communities in the transplanted oysters one month to stabilize, and we used September as the final month, because we lost all samples at the Diedrichsenbank– site in October. The first two principal coordinates of our ordination explained a cumulative percentage of 68.18% and showed a clear grouping of *Vibrio* communities by origin in June. In other words, *Vibrio* communities maintained the signature of their collection site, although oysters were exposed to different site conditions for more than a month, indicating that *Vibrio* communities are somewhat stable on the short term. However, the grouping by origin converged to a clear grouping by sampling site in September (Figure 4), indicating that oyster associated *Vibrio* communities were not population- or genotype-specific for extended periods of time but rather recruit from the surrounding environment, which has also been described for mussels [60]. Overall there was a significant differentiation between the different transplant groups from June and September reflecting temporal changes in community composition (UniFrac significant test after Bonferroni correction: p = 0.02).

### Pathogenicity

Based on controlled infection experiments we were able to assess the pathogenic potential of 70 different *Vibrio* isolates on oyster larvae. There was no mortality in all control treatments. The tested isolates harbored high variation in pathogenicity, ranging from 0 to 100% with a median virulence of 6.25% (Figure 2). About one third of the tested isolates did not induce any mortality. Strain O29w_Aug, identified as member of the *Orientalis* clade, induced 100% mortality. Based on only 16S rRNA sequence similarity this isolate was identified as *V. tubiashii*, which has often been shown to be highly pathogenic to bivalves [61], [62]. In total we found 12 strains that induced more than 30% mortality. In addition, the three most pathogenic strains, that induced mortalities above 50%, were isolated during spawning season, i.e. July–September.

**Figure 5:**
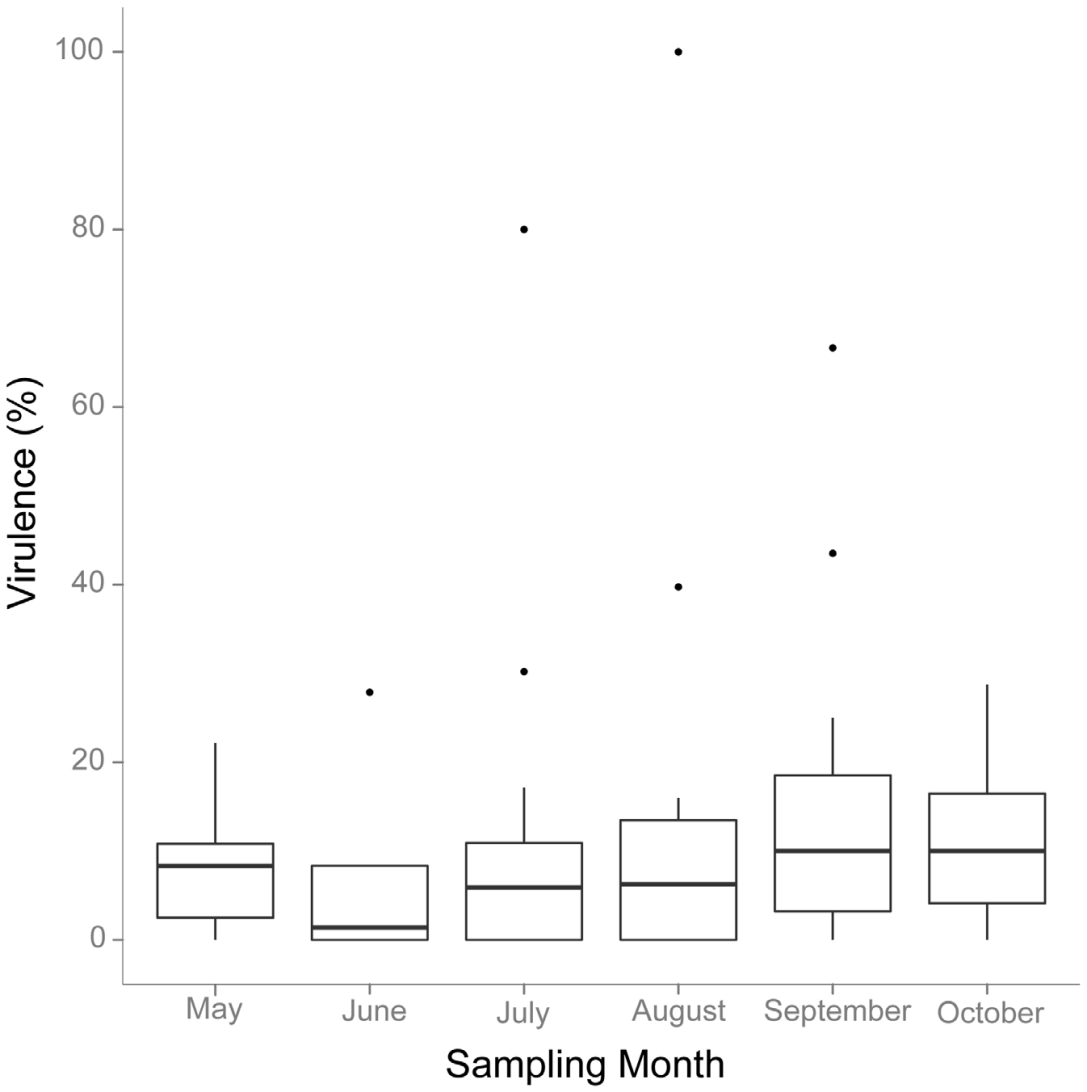
Distribution of induced virulence (%) from May to October 2010. Shown are median virulence values (%) per sampling month, with an interquartile box and a 5 to 95% range. Outliers are marked by black dots.

**Figure 6:**
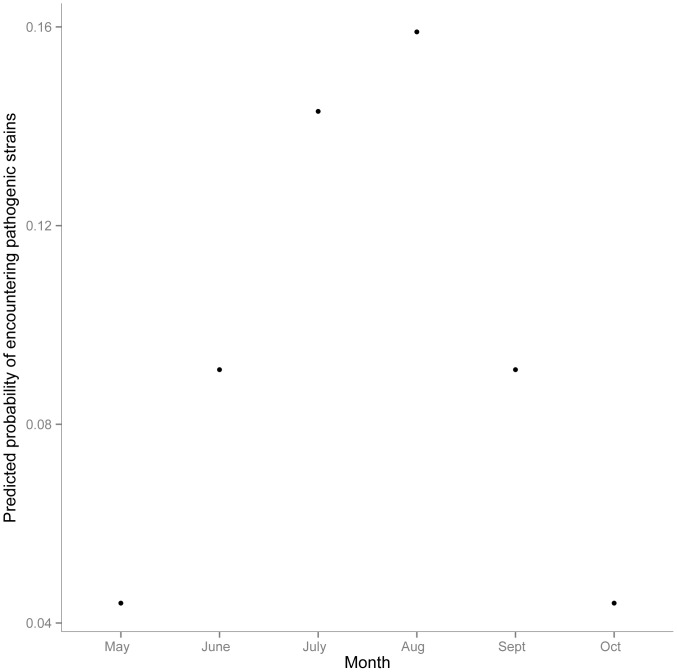
Predicted probability to encounter highly pathogenic strains (i.e. induced mortality >25%) for each sampling month.

On average, the majority of strains within each sampling month displayed low pathogenicity (Figure 5) and there was no significant association between mean virulence per month and temperature (Spearman rank correlation: r = 0.65, p = 0.16). Although diversity correlated well with mean induced virulence per month, we could not find a significant relationship (Chao1 index: Spearman rank correlation: r = 0.77, p = 0.10; Shannon-Wiener Index: Spearman rank correlation: r = 0.64, p = 0.17), which is probably owed to low statistical power associated with N = 6. However, highly virulent strains were exclusively found in summer months especially during spawning season (Figure 5) and we observed a significant correlation between maximum virulence per sampling month and diversity (Chao1 index: Spearman rank correlation: r = 0.89, p = 0.03; Shannon-Wiener Index: Spearman rank correlation: r = 0.75, p = 0.08), which was also reflected in a nearly significant correlation of maximum virulence per sampling month with temperature (Spearman rank correlation: r = 0.77, p = 0.08). The predicted probability of encountering a highly virulent strain increased during summer and was highest in August, i.e. during spawning season where it is on average four times higher than in spring (Figure 6).

## Discussion

The *Vibrio* community in the hemolymph of healthy Pacific oysters *C. gigas* showed strong seasonal variation in diversity, prevalence and virulence of *Vibrio*, with highest values of all parameters coinciding with the spawning season in warmer summer months when mass mortalities usually occur. Environmental temperature therefore seemed to be a major driver for *Vibrio* community dynamics in oyster hemolymph, reflecting patterns observed for open water communities [28], [63]. Although *Vibrio* community dynamics did not immediately react to temperature shifts, we could show with a reciprocal transplant experiment that the *Vibrio* community associated to oyster hemolymph is not persistent over extended periods of time but seems to be recruited from environmental reservoirs (Figure 4).

### Diversity

The structure and diversity of the *Vibrio* community was largely driven by temperature. The diversity of the *Vibrio* community increased with increasing temperatures from May (minimum sea water temperature: 5.9°C) to August (maximum sea water temperature: 21.67°C), followed by a decrease as temperatures dropped in autumn. Low diversities in colder months could have underestimated the true diversity due to our isolation procedure at 25°C, which may have excluded psychotropic strains. We were however mainly interested in sources and reservoirs of potential pathogens that usually impose risk on oysters during warmer summer months.

Members of the *Splendidus* clade (*V. splendidus* and *V. crassostreae*) were predominating and present in every month, followed by members of the *Vibrio* core and the *Orientalis* clade, which were only present in the warm summer months. In general, bacterial communities of Pacific oysters [26] as well as coastal waters [64] are often dominated by *V. splendidus* related strains when temperatures are below 20°C, whereas members of the *Vibrio* core are most prevalent when sea water temperatures are above 20°C [65]. Similarly, *Vibrio* communities in the hemolymph of the spider crab *Maja brachydactyla* were dominated by species belonging to the *Splendidus* clade in areas with water temperatures below 20°C, while members of the *Vibrio* core, the *Orientalis* and the *Splendidus* clade dominated equally in areas where water temperatures were around 21°C [38]. This suggests that temperature dependent composition of different *Vibrio* communities follows similar rules in host associated microbiota as well as open water communities [28], [65], [66]. Our lag-analysis, on the other hand showed that *Vibrio* communities in Pacific oysters are largely inert to quick temperature shifts and that the effect of temperature on community composition is strongest when integrated over longer periods of time, indicating that these communities are stable for relatively long periods of time.

### Abundance

The abundance of cultivatable *Vibrio* decreased throughout the summer season before it peaked just after the spawning season, indicating that abundance within oyster associated communities did not react directly to water temperatures (Figure 1). This is in contrast to open water abundances of *Vibrio* that usually correlate positively with water temperature [28], [65], [67]. Unfortunately, we do not have any data on *Vibrio* abundance in the open water column here, but *Vibrio* abundance in the North Sea were also shown to correlate with temperature [63], indicating that there may be a mismatch between open water abundance and abundance within oyster hemolymph.

Deviations from temperature correlated *Vibrio* population sizes may also be caused by several other environmental factors, such as salinity or nutrients [63]. In algal blooms (March–May), *Vibrio* abundances were correlated to Chlorophyll a, indicating that high algal abundance might provide high amounts of nutrients [63]. Consumption of *Vibrio*-loaded algae might therefore explain the high *Vibrio* abundances observed at the beginning of our study (May). Similarly, the steep increase after the spawning season might be attributable to high take-up of larvae that were previously colonized by *Vibrio*.

Additionally, part of the mismatch between environmental and oyster hemolymph abundances can be caused by the interaction between *Vibrio* and host condition or host immune response. In a previous infection experiment we observed that there was no difference in mortality between gravid and pre-spawning oysters when infected with several *Vibrio* strains [22]. Post-spawning oysters, on the other hand, suffered from high mortality rates resulting from insufficient clearance of pathogens due to an impaired phagocytic ability of hemocytes. Therefore, the strong increase in CFU in oyster hemolymph may reflect the impaired ability of oysters to keep *Vibrio* abundance low just after spawning.

### Persistence

The natural microbiota of oysters can be resistant to environmental perturbation and can persist during depuration processes [68]. Similarly, under ambient conditions the total microbial community composition depended on host genotype in the oyster beds investigated here [40] and the genetic differentiation observed among the oyster beds, may suggest that also *Vibrio* communities of oyster hemolymph assemble in a bed specific manner resembling host genetic differentiation. Our reciprocal transplant experiment could disentangle effects of spatial origin or host genetic factors from environmental effects. The Principal Coordinate Analysis (PCoA) revealed a clear grouping of the different transplant groups separating communities based on origin, and thus potentially host genotype, in June. At the end of the sampling period in September oyster bacterial communities converged by experimental site rather than origin (Figure 4). These findings are in concert with a study on the metapopulation structure of *Vibrionaceae* of blue mussels and crabs suggesting a neutral population structure without host preferences [60]. The spatial convergence over time observed in the present study therefore suggests that similarly oyster associated *Vibrio* community is not stable within hosts, and rather depends on *Vibrio spp.* taken up from the surrounding environment.

### Pathogenicity

Our infection experiment demonstrated that *Vibrio* strains isolated from healthy adult Pacific oysters have the potential to kill oyster larvae under controlled experimental conditions. In total we found 12 pathogenic strains killing more than 30% of the oyster larvae out of which the most virulent strain O29w_Aug, identified as member of the O*rientalis* clade, killed 100% of oyster larvae. Although we are not aware of any study on the pathogenicity of *V. orientalis*, there are reports of several strains from the *Orientalis* clade (e.g. *V. tubiashii*) that have a high pathogenic potential for bivalves [61], [62]. The majority (8 out of 12) of the strains that induced high mortalities were isolated during spawning season, i.e. July–September when the probability of encountering such strains was highest (Figure 6). Since highly virulent strains may have the potential to cause epidemic mass mortalities, the observed increase in virulence during summer reflects the worldwide summer mortality patterns of larvae, spats and adult oysters [13], [14], [15], [16], [69].

Oyster summer mortalities affecting all life-stages of *C. gigas* are the result of a complex interaction of adverse environmental factors (e.g. high temperature), host physiological condition and genetics, and pathogens, such as Ostreid herpesvirus 1 and *Vibrio* spp. [8], [9], [10], [18], [20], [21], [22], [33], [70], [71]. This complex interaction affecting all life-stages makes it difficult to assess the risk of mortality. Here, we concentrated on *Vibrio* strains that showed the ability to colonize the hemolymph of adult oysters, and its pathogenic potential to larvae. Our interest on oyster larvae was due to several reasons. First, larvae occur only at high temperatures during summer month, which coincides with a peak in *Vibrio* abundance in the study area [63], [72]. Hence, any time larvae occur, they are at high risk of *Vibrio* infection. Therefore we also isolated the *Vibrio* strains at 25°C, representing high summer temperatures, and hence allow the detection of pathogenic *Vibrio* that are present in warm waters. Second, larvae mortality is one of the main drivers for stock recruitment. Therefore, assessing the risk of mortality on early life-stages is essential to draw conclusions about the impact of potential disease outbreaks on local oyster populations only if the pathogenicity of local strains is known. Third, although oyster hemolymph seems to be relatively isolated from the surrounding waters, we demonstrated in the present study, that oyster associated *Vibrio* community is to a large extend influenced by the surrounding environment. Therefore we could show that the isolated strains are likely to be encountered by larvae, which are in direct contact with the water column and in close proximity to the adult oysters.

By characterizing the *Vibrio* community of healthy Pacific oysters, we were indeed able to isolate highly pathogenic strains to larvae and could show, that the probability to encounter those strains is highest in summer, during spawning season. However, overall the amount of highly pathogenic strains as well as the probability to encounter them was relatively low. Nevertheless, the chance of observing highly virulent strains that could cause such mass mortalities was higher in more diverse communities suggesting that diversity can be used partly as an indicator for the maximum virulence to be expected at a given sampling time point. Since diversity was correlated to mean monthly temperature, temperature driven shifts in the composition of the *Vibrio* community, as could be expected with a projected increase in mean water temperature in the future, could increase the risk of the presence of highly pathogenic strains during warm summer months contributing to potential mass mortalities. On the other hand, larval mortality rate induced by certain *Vibrio* strains may be a weak predictor of juveniles and adults mortality. Indeed, some strains from the present experiment have also been tested on adults [73] and virulence differed substantially between life-stages with an overall higher impact on adult oysters than on larvae. Nevertheless, adults may act as reservoir of pathogenic *Vibrio* strains to earlier life stages and are hence important for the transmission and spreading of these pathogens.

### How will rising temperatures affect the oyster associated Vibrio community?

*Vibrio* diversity and abundance increased substantially during and after spawning. A single shellfish can contain between 100 to 1000 strains [74], however mortality can result from a single strain in a heterogeneous population found in shellfish [75]. Containing highly diverse repertoires of *Vibrio* spp. therefore seems to increase the chance of any such highly virulent strain being present in the animal. Furthermore studies using simultaneous injection of several strains observed increased virulence [10]. Such synergistic effects between *Vibrio* strains are yet more likely to happen when *Vibrio* diversity is high like in summer months. Although oysters in the northern parts of the North Sea never experienced summer disease outbreaks, pathogenic *Vibrio* strains are present in the ambient microbiota and could react to changing environmental conditions. Average water temperatures during summer months in the investigated area are about 18°C, while oyster summer mortality appears to occur when temperatures exceed a threshold of 19°C to 20°C [21]. We performed the experimental infections with the isolated *Vibrio* strains at 21°C, which is higher than the average water temperature observed during our study. The most virulent strains found here have been assigned to the *Orientalis clade* and the *Vibrio* core, but we found substantially fewer *Vibrio* strains associated to the *Orientalis clade* and the *Vibrio* core than similar studies in France during an episode of extensive mass mortalities [26]. Since members of the *Vibrio* core dominate when sea water temperatures are above 20°C [65], we might expect a shift from a *V. splendidus* dominated community to a community that is dominated by members of the *Vibrio* core and the *Orientalis* clade, when temperatures rise as predicted by current climate change models. Such a temperature dependent community shift can then result in an increase of diversity, abundance and virulence of the oyster associated *Vibrio* community given that predicted probability to encounter pathogenic *Vibrio* spp. also increases with increasing temperature.

### Conclusion

With the present study we could demonstrate that persistence, dynamics, and diversity of the *Vibrio* community in the hemolymph of Pacific oysters underlies strong seasonal effects. The likelihood to encounter pathogenic strains that represented a considerable proportion (∼18%) of the strains with the ability to colonize oyster hemolymph was highest in warm summer month during and after spawning season. By means of a reciprocal transplant experiment we could show that *Vibrio* spp. were permanently present in hemolymph microbiota, but formed an unstable, dynamic community that was recruited from the surrounding environment.

As summer mortalities of *C. gigas* have been observed solely 130 km southward, ongoing research focusing on the study of the *Vibrio* ecology including methods to predict the pathogenic potential of a strain are pivotal in this area, where oyster aquaculture is prominent. Continuously monitoring the key parameters of diversity, abundance and pathogenic potential of *Vibrio* from all potential reservoirs (sediments and open water) as well as their affected targets (oyster hemolymph and larvae), can therefore help to detect first signs of summer mortality heralded by a change in the natural community structure. The ability to identify epidemiological patterns linked to bacterial community structure and specific dynamics of candidate pathogens will be a high priority, especially when infectious diseases are predicted to increase with global warming [76].

## Supporting Information

Figure S1 **Correlation between colony forming units and OD values at 550 nm for 10 selected** ***Vibrio*** **strains of low, intermediate, and high virulence.** An OD value of 1 at 550 nm corresponds to 4.63–5.24×10^8^ CFU/ml.
(TIF)

Table S1 **Sequences of all primers used for genotyping the bacteria of the genus** ***Vibrio*** **spp. (** ***pyrH*** **,** ***GyrB*** **, 16S rRNA).**
(DOCX)

Table S2 **Reference** ***Vibrio*** **sequences used for the phylogeny.** The references sequences were aligned to the sequences of the particular gene (*16S rDNA*, *pyrH*, *GyrB*).
(DOCX)
