# Supplementary figures and images for: Persistence, Seasonal Dynamics and Pathogenic Potential of Vibrio Communities from Pacific Oyster Hemolymph
Source: PLoS One. 2014 Apr 11;9(4):e94256. doi: 10.1371/journal.pone.0094256 (PMC3984124; doi:10.1371/journal.pone.0094256)

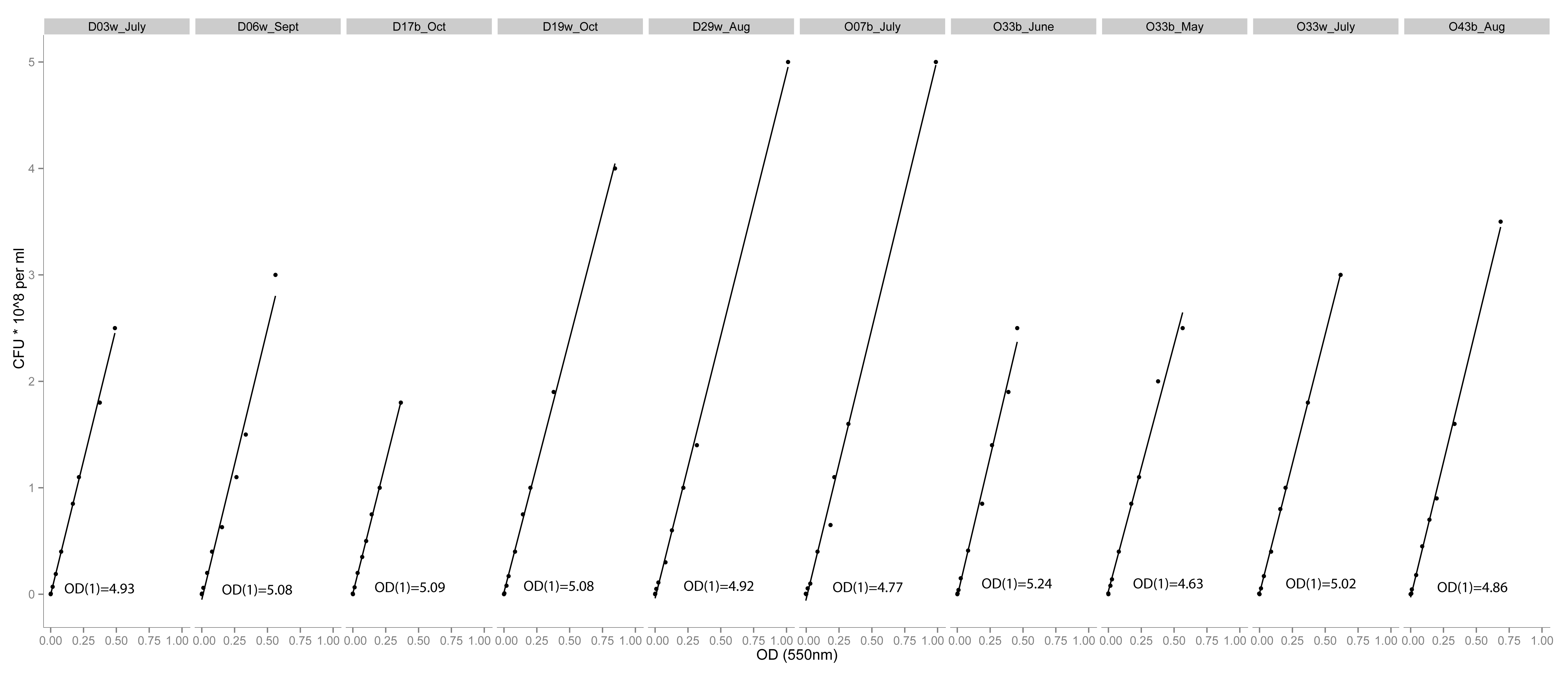

Supplement: Figure S1 — Correlation between colony forming units and OD values at 550 nm for 10 selected Vibrio strains of low, intermediate, and high virulence. An OD value of 1 at 550 nm corresponds to 4.63–5.24×10^8^ CFU/ml. (TIF) [file pone.0094256.s001.tif]
