# Supplementary material for: Persistence, Seasonal Dynamics and Pathogenic Potential of Vibrio Communities from Pacific Oyster Hemolymph
Source: PLoS One. 2014 Apr 11;9(4):e94256. doi: 10.1371/journal.pone.0094256 (PMC3984124; doi:10.1371/journal.pone.0094256)
Supplement: Table S1 — Sequences of all primers used for genotyping the bacteria of the genus Vibrio spp. ( pyrH , GyrB , 16S rRNA). (DOCX) [file pone.0094256.s002.docx]

**Table S1**

| **Gene product** | **Primer** | **Sequence (5’- 3’)** |
| --- | --- | --- |
| Uridylat Kinase (*pyrH*;552nt) | pyrH-02-R | GTRAABGCNGMYARRTCCA |
|  | pyrH-04_F | ATGASNACBAAYCCWAAACC |
| Gyrase subunit B(*GyrB*; 939nt) | up1 E_F | GAAGTCATCATGACCGTTCTG |
|  | up2A_R | AGCAGGGTACGGATGTGCGAGCC |
| 16S rRNA | 16S 27-F | AGAGTTTGATCATGGCTCAG |
|  | 16S 1492-R | TACCTTGTTACGACTT |
