# Supplementary material for: Persistence, Seasonal Dynamics and Pathogenic Potential of Vibrio Communities from Pacific Oyster Hemolymph
Source: PLoS One. 2014 Apr 11;9(4):e94256. doi: 10.1371/journal.pone.0094256 (PMC3984124; doi:10.1371/journal.pone.0094256)
Supplement: Table S2 — Reference Vibrio sequences used for the phylogeny. The references sequences were aligned to the sequences of the particular gene (16S rDNA, pyrH, GyrB). (DOCX) [file pone.0094256.s003.docx]

**Table S2**

| **Name** | **16s** | **PyrH** | **GyrB** |
| --- | --- | --- | --- |
| *V. splendidus* | AB038030 | EU118241.1 | EF380261.1 |
| *V. cholerae* | X76337 | FM202582.1 | FM202624.1 |
| *V. parahaemolyticus* | X74720 | GU266286.1 | FM202617.1 |
| *V. cyclitrophicus* | DQ481610.1 | GU378520.1 | DQ164536.1 |
| *V. gigantis* | EU579451.1 | EU871951.1 | AJ577817.1 |
| *V. crassostreae* | NR_044078 | EU871948.1 | AJ582797.1 |
| *V. harveyi* | AY332564.1 | FM202542.1 | FM202583.1 |
| *V. campbellii* | JX442511.1 | FM202561.1 | FM202603.1 |
| *V. alginolyticus* | X74690 | FM202578.1 | FM202620.1 |
| *V. natrigens* | JN641992.1 | FM999815.1 | FM999818.1 |
| *V. lentus* | NR_028926.1 | EU871959.1 | AM162564.1 |
| *V. fischeri* | EF667055.1 | EU185900.1 | EU185872.1 |
| *V. vulnificus* | HM996973.1 | EU118257.1 | AY705492.1 |
| *V. orientalis* | NR_026127.1 | EU118243.1 | EF380260.1 |
